# The effect of area of residence over the life course on subsequent mortality

**DOI:** 10.1111/j.1467-985X.2008.00581.x

**Published:** 2009-06

**Authors:** Alastair H Leyland, Øyvind Næss

**Affiliations:** Medical Research Council Social and Public Health Sciences UnitGlasgow, UK; University of Oslo and Norwegian Institute of Public HealthOslo, Norway

**Keywords:** Cross-classified models, Life course epidemiology, Multilevel models, Multiple-membership models

## Abstract

Life course epidemiology concentrates on the contribution that social or physical exposures have across the life course on adult health. It is known that the area of residence can affect health, but little is known about the effect of the area of residence across the life course. We examine the contribution that area of residence in 1960, 1970, 1980 and 1990 made on subsequent mortality for 49736 male inhabitants of Oslo in 1990. We compare the performance of multiple-membership and cross-classified multilevel models on these data with a correlated cross-classified model that was developed for this.

## 1 Introduction

A recent focus of chronic disease epidemiology has been in how exposures across the whole life course may influence health in adult life (Kuh and Ben-Shlomo, 2004; Pickles *et al.*, 2007). Many chronic conditions such as cancers and cardiovascular diseases have long latency periods, meaning that they develop over time (Rose, 1982). Various studies have indicated that exposures in early life may be involved in the initiation of disease processes. Such exposures during critical periods of fetal life and childhood growth may have long lasting effects on risk of disease in adults and suggest time lags between exposure and the clinical manifestation of disease (Lynch and Davey Smith, 2005). This approach emphasizes that the timing of an exposure may be important for the development of biological subsystems, such as anatomical structure and physiological function, and for social transitions from childhood to adulthood, such as migration from the parental home, the establishment of one's own residence and exiting the labour market. Similarly, many risk factors in adult life tend to track over the life course, resulting in increased risk depending on the duration of exposure, such as smoking, dietary habits, physical exercise and environmental pollutants. These determinants may cluster spatially and accumulate longitudinally through the life course. The life course approach explicitly emphasizes that, to acknowledge fully the patterning of disease risk in adulthood, factors from all stages of the life course need to be taken into account. This means that the life course approach can only be applied to those who survive until adulthood.

A large body of literature exists examining the relationship between the place of residence and health and the reasons why such a relationship may exist. Environmental influences on health extend beyond the obvious physical factors such as a lack of clean water or exposure to pollutants (Marmot, 2005) to socio-economic factors (Macintyre *et al.*, 1993). With health (or ill health) patterned by socio-economic circumstances, the neighbourhood or community of residence is often put forward as a potential mechanism linking the two (Diez Roux, 2001). The uptake of multilevel modelling in epidemiological and public health research has been key in the investigation of ways in which an individual's context can influence health (Pickett and Pearl, 2001). The difficulty in inferring causality from multilevel observational studies of neighbourhood effects, i.e. in showing that it is residing within a particular area that leads to ill health, has been identified as a challenge for future research (Oakes, 2004). One potential solution to this problem that has been proposed is to measure contextual exposures repeatedly—including the area of residence—in addition to measuring individual exposures across the life course (Subramanian, 2004). There has, however, been a distinct lack of research into the extent to which the area of residence at different stages of the life course may influence health, possibly because of a paucity of suitable data. Strachan *et al.* (1995) analysed the effect of place of residence in 1939 (at age 0–16 years) and 1971 on death from ischaemic heart disease and stroke between 1971 and 1988. They divided England and Wales into 14 regions and used a fixed effects model to separate the effect of region at the two time points. Using an extension of the same data, Curtis *et al.* (2004) considered the separate effects of the place of residence in 1939 and 1981 on death between 1981 and 1991 and (among those still alive) on self-reporting of limiting long-term illness in 1991. However, the cross-classified multilevel models that they used did not include area of residence at each time point *per se* but rather a combination of region (England and Wales was split into four regions) and type of area. Chandola *et al.* (2005) examined the influence of area of residence between 1991 and 1998—yearly—on physical and mental health functioning in 1999. They used a multiple-membership multilevel model with area defined by electoral wards—fairly small areas containing between one and 10 households in the sample that was used. Næss *et al.* (2008) used data from the Oslo mortality study, based on individuals who were resident in Oslo in each of 1960, 1970, 1980 and 1990, to examine area effects on mortality from 1990 to 1998. They used a cross-classified multilevel model to estimate effects at a small area level.

We are interested in reanalysing the Oslo mortality study data with a view to determining the extent to which the area of residence at different stages of the life course impacts on subsequent mortality. So for someone who was aged between 30 and 39 years in 1990 we want to ascertain the relative importance of the area of residence in each of 1960 (aged 0–9 years), 1970 (aged 10–19 years), 1980 (aged 20–29 years) and 1990. If we can estimate a variance that is associated with the area of residence at each time point then we can partition the total variance due to area of residence (at these four times) into that attributable to each time point. Multilevel models are commonly used to partition variance and there is a clear hierarchical structure to the data, with individuals living in one area at each time. If we find one time point to be more important than the others—i.e. it is associated with a larger variance—then there is a suggestion that this is a critical period for contextual influences on development. If, in contrast, we find a suggestion that all time points are of equal importance then we can view this as some evidence that risk is being accumulated across the life course.

There remains a question about what model is suitable for the analysis of the effect of area over the life course. The two previously used approaches—multiple-membership models and cross-classified models—make different assumptions about how the effect of an area on individual health changes over time. Both models have their disadvantages. In this paper we describe the data set and then detail various models that could be used to model the data. In particular, we consider simple hierarchical models that use information from area of residence at just one time point, multiple-membership models and cross-classified models. We also show how the multiple-membership model can be extended for such data to enable empirical estimation of the importance that is attached to an area at each time point, and how multiple-membership models and cross-classified models can be combined to allow correlations between areas over time to vary between 0 and 1. We then present the results of fitting these models to the data and finish by drawing some conclusions about the use of such models in epidemiological research, as well as drawing substantive conclusions regarding these data.

## 2 Data set

Census information from 1960, 1970, 1980 and 1990 was linked for individuals to the death register (including deaths from 1990 to 1998). The study population was all male inhabitants of Oslo on January 1st, 1990, who were aged 30–69 years: a total of 122951 individuals. There were relatively few deaths among women in the younger age groups and we have restricted our analysis to men. To study the residential history of people in Oslo, only those who had been resident at all four censuses were included: a total of 50860 men. Immigrants to Oslo during this period had an age-adjusted mortality rate of 101 (per 10000 person-years). Of the cohort selected, people with missing data on area of residence at any of the censuses were excluded—a total of 1124 individuals with an age-adjusted mortality rate of 211 (per 10000 person-years). The remaining included population (*n*=49 736) had an age-adjusted mortality rate of 111 (per 10000 person-years).

Life course epidemiology—and in particular the study of chronic diseases—requires the analysis of the contributions of risk factors at all stages of the life course (Kuh *et al.*, 2003; Kuh and Ben-Shlomo, 2004). In this way we can gain an understanding of the natural history of a disease. For this reason we have excluded deaths before 1990; the study population therefore has an inevitable bias towards healthy survivors. The areas of residence are administrative areas used for the organization of elections. The residential area code was known for the studied population at each of the four censuses. Areas were first given codes in 1960. In 1970 a new coding system was introduced which had a higher resolution, but for continuity we applied the 1960 area codes to each census. Most of the new area codes that were used from 1970 onwards were geographically nested within the 69 area codes from 1960. For a few places, such as industrial sites, the borders did not coincide exactly and the coding of an individual to an area was based on correspondence between street addresses.

The proportion of the population who migrated between censuses changed with age, but the overall pattern was similar for all cohorts. Young people tended to be more mobile, with migration reaching a peak during the 10 years after the census in which they were aged 20–29 years. In the youngest cohort, 61% of the population included moved from one area to another between the 1980 and 1990 censuses (those in this cohort were aged 20–29 years in 1980); the same percentage of the cohort of those aged 40–49 years in 1990 migrated between the 1970 and 1980 censuses. Of those aged 50–59 years in 1990, 68% migrated between the 1960 and 1970 censuses. Migration levels then decreased as those in each cohort aged; the lowest migration level (15%) was seen between the 1980 and 1990 censuses for those aged 60–69 years in 1990. The lower level of migration means that in this cohort there will be less power to detect differences between the effect of place of residence in 1980 and 1990. The median size of population in each area decreased over time with a corresponding increase in heterogeneity of size (as measured by the standard deviation of the populations). Areas with different sizes had similar age-adjusted mortality at each time point. More detail of the data is provided elsewhere (Næss *et al.*, 2008).

We illustrate the format of the data in Table 1 by providing details of the residential history or migration patterns of five hypothetical individuals, all of whom lived in the same area (area 1) at the time of the 1960 census. The first person also lived in area 1 at the three subsequent censuses. (Note that he may have moved out of this area and subsequently returned between censuses; however, we have no information about such movements and so, for this person, we assume that he lived in the same area at all times.) Person 2 moved from area 1 to area 2 between 1970 and 1980. Person 3 moved from area 1 to area 2 between 1960 and 1970 and then returned to area 1 between 1980 and 1990. Person 4 also moved from area 1 to area 2 between 1960 and 1970, but moved to a third area (area 3) between 1980 and 1990. Person 5 moved to a different area between every census. We may expect the effect of residential area on health to differ for these people for various reasons. Firstly, the five people lived in four different areas by the time of the 1990 census and we might reasonably expect the different areas in 1990 to have differential effects on mortality between 1990 and 1998. Secondly, the length of time that each person lived in area 1 varied; person 1 lived there at all four censuses but person 4 and person 5 each lived there for just one census. If something about living in this area is damaging to an individual's health then we would expect the length of exposure to that area to be important. Finally, the time at which people lived in the same area does not always coincide. So person 2 and person 3 both lived in areas 1 and 2 at two of the censuses, but in 1970 and 1990 they were living in different areas. The effect of living in area 1 on each person's health may differ if the area itself changed between 1970 and 1990—e.g. if a polluting source started to operate—or if the different ages at which individuals lived in an area meant that they had differential susceptibility to their environment (e.g. in terms of the development of organs in early life or the time spent in the area of residence following retirement).

**Table 1:** Example of migration histories over four censuses for five individuals

*Person*	*Areas of residence in the following years:*			
	*1960*	*1970*	*1980*	*1990*
1	1	1	1	1
2	1	1	2	2
3	1	2	2	1
4	1	2	2	3
5	1	2	3	4

## 3 Modelling area effects

The data related to men in Oslo and were broken down into four cohorts according to age in 1990: those aged 30–39 years (*n*=12 529), 40–49 years (*n*=11 357), 50–59 years (*n*=9967) and 60–69 years (*n*=15 883). The four cohorts were analysed separately. The mean population in each area ranged from 144 for those aged 50–59 years to 230 at ages 60–69 years. Differences in the size of the study population in each age group—including the potentially surprising finding that the population is larger for those aged 60–69 years in 1990 than for any of the other age groups—reflects a combination of differences in the size of the original birth cohorts, differential probabilities of migration out of Oslo and different survival probabilities into the age of follow-up. The outcome that was considered was death at any stage during the follow-up. The response that was used was therefore binary (1, died; 0, did not die). Model estimation was by Bayesian Markov chain Monte Carlo sampling, necessitating the specification of prior distributions on parameters (see Section 4). All models were fitted using WinBUGS version 1.4.1 (Spiegelhalter *et al.*, 2004) running two parallel chains, discarding the first 10000 replicates and basing inference on the next 100000 for each chain.

We model the probability of death at any stage during the follow-up period from 1990 to 1998; mortality is sufficiently rare in all four cohorts for the odds ratios to approximate hazard ratios. We consider several ways of modelling the data; in this section we present the algebraic description of the models together with the strengths and weaknesses of each. The models that were considered come under the multiple-membership multiple-classification models that were developed by Browne *et al.* (2001). The notation that was developed by them presupposes that we can distinguish between a cross-classified and a multiple-membership data structure; since we wish to examine both (together with a combination of the two) we have used slightly different notation but refer to Browne *et al.* (2001) where possible. We denote the areas in which individual *i* lived by area60(*i*) in 1960, area70(*i*) in 1970, area80(*i*) in 1980 and area90(*i*) in 1990. So for person 1 in Table 1 we have area60(1)=area70(1)=area80(1)=area90(1) (=1) whereas for person 2 we have area60(2)=area70(2) (=1) and area80(2)=area90(2) (=2). Then for all models we assume a Bernouilli distribution for the response *y*_*i*_ for individual *i*, i.e.
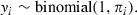
(1)

We assume a logit link from the probability *π*_*i*_, related to a vector of characteristics 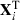, specific to individual *i* through a vector of fixed parameters ***β***, and to the random effects denoting the area of residence at each census. We have restricted covariate information to two dummy variables indicating to which 5-year age group individual *i* belongs. For example, when modelling the 30–39 years cohort in 1990 we have a dummy variable *x*_0*i*_ which takes the value 1 if individual *i* was aged 30–34 years in 1990 and 0 otherwise, and a variable *x*_1*i*_=1−*x*_0*i*_ which takes the value 1 if individual *i* was aged 35–39 years in 1990 and 0 otherwise. We then have a vector of fixed parameters ***β***=(*β*_0_,*β*_1_)^T^. (This is an alternative parameterization to that with an overall intercept and a dummy variable for one age category which reduces the correlation between the two fixed parameters being estimated.)

We assume an area effect 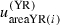 of living in area areaYR(*i*) in year YR on subsequent mortality. The full model can then be written as 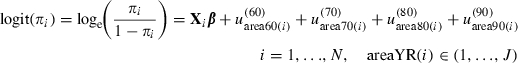
(2) or in matrix notation as 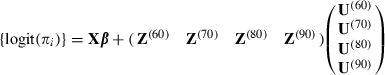
(3) where **X**={**X**_*i*_} is the fixed part design matrix that is formed by stacking the individual characteristics **X**_*i*_, **U**^(YR)^ is the vector of the residuals for areas in year YR, 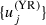, *j*=1,…,*J*, and **Z**^(YR)^ is the random-part design matrix that identifies the area in which an individual lived in year YR. The four residuals for any particular area *j* corresponding to the four different census years are assumed to have a joint normal distribution, with the exact composition of the dispersion matrix **Σ** of order 4 being what differentiates between the models that are presented below, 
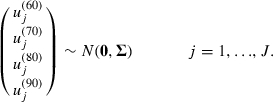
(4)

Note that we still assume that the residuals for different areas are *a priori* uncorrelated, regardless of the year. This means that the residuals for areas 1 and 2, for example, would have a joint distribution given by
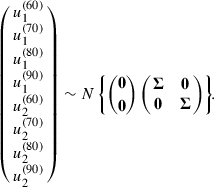
(5)

### 3.1 Model A^(60)^, A^(70)^, A^(80)^, A^(90)^: separate two-level models including the effect of area of residence at a single year

We include the effect of the area of residence in a single year only. Model A^(60)^, for example, considers the effect of the area of residence in 1960 on subsequent mortality. We can rewrite equation (2) as 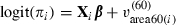
(6) where 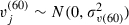
(7) by writing 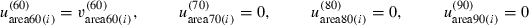
(8) so that 
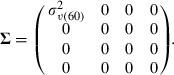
(9)

Throughout this section we illustrate the variances of logit(*π*_*i*_) that are implied by the various models for person 1 and person 5 in Table 1 together with the covariance between them. Since all five people lived in area 1 in 1960, the variances and covariances are all given by 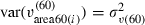. This is a simple model which would be appropriate if the area of residence at one time point only were known (e.g. the area of birth or the most recent area of residence). If there were no migration between areas between censuses then this model would also be appropriate; however, since the proportion of the population that is resident in the same area in all the censuses varied between just 14% (those aged 40–49 years in 1991) and 38% (aged 60–69 years), this model does not make full use of the available data. We can compare the four models to elucidate information regarding the importance of each year by using either the area variance or the deviance information criterion (DIC) (Spiegelhalter *et al.*, 2002). (The DIC is a Bayesian measure of model fit that is penalized for the effective number of parameters in the model.) However, when considering only residence at one time in each model we cannot partition the variance and therefore cannot quantify the extent to which area of residence at each time point contributes to mortality. (We cannot, for example, make a statement that is based on these models such as ‘more than half of the total area variation is associated with the area of residence in year *X*’.)

### 3.2 Model B: multiple-membership model

Multiple-membership models are used when an individual is simultaneously a member of more than one higher level unit. These models have been developed in an educational context in which pupils may receive their education from more than one school (see for example Goldstein (2003) and Fielding and Goldstein (2006)), although they are also appropriate when group membership (such as school or class) is unknown (Hill and Goldstein, 1998). More recently they have been applied to the problem of assessing the effect of area of residence on health, taking population mobility into account (Chandola *et al.*, 2005). In the case of the Oslo mortality study each individual has resided in between one and four areas (and is therefore a ‘member’ of between one and four areas). In Table 1 person 1 is an example of someone who lived in just one area, person 2 and person 3 lived in two areas, person 4 in three areas and person 5 in four areas. Multiple-membership models require the specification of weights associated with each higher level unit; in the absence of further information, and in line with earlier research (Chandola *et al.*, 2005), we can assign weights that are proportional to the number of times that an individual was observed to live in each area. In the notation of Browne *et al.* (2001) we rewrite equation (2) as 
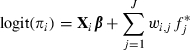
(10) where 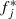 is the effect of living in area *j* on subsequent mortality, the *w*_*i*,*j*_ are the weights that are associated with area *j* for individual *i* and which sum to 1 for each individual, 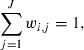
(11) and the area effects are assumed to be independently and normally distributed 
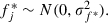
(12) (This notation is detailed in Fielding and Goldstein (2006).) Note that the multiple-membership classification specifies the areas in which a person lived, but not at what census he lived there. Everyone had an area of residence at all four censuses; setting the weights *w*_*i*,*j*_ to 0 for those areas in which individual *i* did not live at any time means that equation (10) can be rewritten as 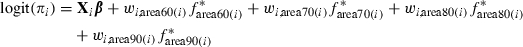
(13) with *w*_*i*,areaYR(*i*)_=0.25 for each census. The number of distinct areas can range from 1 to 4. For example, from Table 1 we can see that person 1 lived in just one area, person 2 and person 3 each lived in two areas and person 5 lived in four areas. The total contribution to equation (13) from the area of residence for person 1 would then be 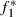 and would be 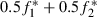 for both person 2 and person 3. We can then simplify equation (13) by writing 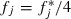, meaning that we can rewrite equation (13) as 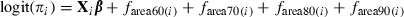
(14) where 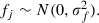
(15)

This can in turn be expressed in terms of equation (2) by writing 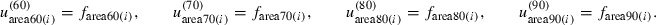
(16)

We also note that if areaYR^1^(*i*)≠areaYR^2^(*i*) for two different census years YR^1^ and YR^2^ that are both elements of {60,70,80,90} then as in distribution (5) the covariance of *f*_areaYR^1^(*i*)_ and *f*_areaYR^2^(*i*)_ is 0. However, where areas are common to censuses we have areaYR^1^(*i*)=areaYR^2^(*i*), and random effects are possibly repeated, this covariance term is 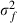. Here we are effectively dealing with the same random effect and so the covariance is equal to the variance. Thus in the framework of equation (2)
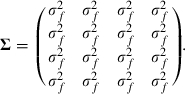
(17)

This model makes the simplifying assumption that area effects are constant over time. This means that the effect of area *j* on mortality between 1990 and 1998 is assumed to be the same regardless of whether an individual lived in that area in 1960 or 1990, i.e. without regard to how the circumstances of the area might have changed or how the effect of an area may differ at different stages of the life course. Taking into account the absence of correlation between different areas, we can see that the variance arising from the area effects will differ according to the length of time that is spent in the same area. Returning to Table 1, the area variance in logit(*π*_*i*_) for person 5 would be 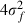 whereas that for person 1 would be 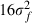, the higher variability for someone living in the same area at all censuses reflecting the covariances in equation (17). The covariance between logit(*π*_*i*_) for person 1 and person 5 that is induced by the fact that they both lived in area 1—person 1 at four censuses, and person 5 at one census—is given by 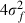.

### 3.3 Model C: unconstrained multiple-membership model

The weighting scheme that is used for the multiple-membership model (model B) makes the assumption that the area of residence is of equal importance at all stages of the life course (since 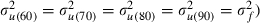. We can relax this assumption by allowing the weights to be proportional to the square root of the variability in the data. We adapt equation (2) by writing 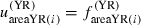 where the variance of the area effects differs between censuses: 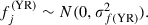
(18)

As with the multiple-membership model that was described in Section 3.2, we maintain a correlation of 1 between area effects in different years by constraining the covariances such that 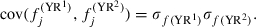
(19)

This gives 
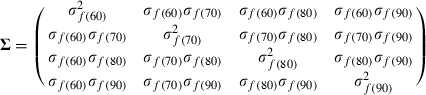
(20) where 
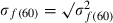
 etc. The variance of logit(*π*_*i*_) for person 1 in Table 1 is now given by 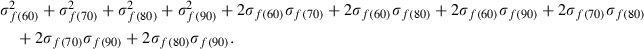


For person 5 the variance is 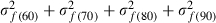 and the covariance between the two is 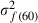. This model can be expressed in the form of a conventional multiple-membership model by writing 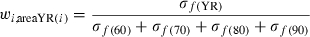
(21) in equation (10) or (13) if individual *i* lived in area areaYR(*i*) in year YR and is 0 otherwise. This ensures that the weights for each individual sum to 1 as in equation (11). The variance of logit(*π*_*i*_) from equation (10) is given by 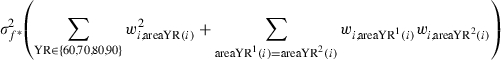 where (*YR*^1^≠*YR*^2^) ∈ {60,70,80,90}. Writing 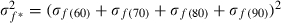 it can be seen that the variances are the same as for the unconstrained multiple-membership model. Such a weighting system—with the weights essentially being derived from the data on the basis of the variances as described above—is an alternative to the exploration of the effects of different weighting systems that were described by Goldstein *et al.* (2007). Although this model now enables an estimation of the contribution of area of residence at each stage of the life course to mortality, it is still based on the assumption that area effects are perfectly correlated over time. Although we might expect some positive correlation—areas that are more damaging to health than average are unlikely to become areas that benefit health over a short time period—it is likely that there will have been some change to the relative merits of areas over a period of 30 years.

### 3.4 Model D: cross-classified model

Cross-classified models are used when there is no strict hierarchical structure to higher level units (Goldstein, 1994). Such models typically comprise individuals who are nested within a cross-classification of two differing hierarchies such as students nested within a cross-classification of schools and neighbourhoods. In our model we have four classifications relating to the areas at each census year, i.e. we have four classifications referring to what is essentially the same hierarchy (the 69 areas in Oslo). We adapt equation (2) by writing 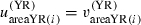 where the variance of the area effects differs between censuses: 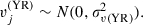
(22)

This model is distinguished from the unconstrained multiple-membership model—model C—by the assumption that the residuals are independent. So we have 
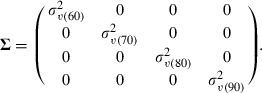
(23)

For both person 1 and person 5 in Table 1 the variance of logit(*π*_*i*_) is 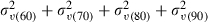, and the covariance between the two is 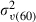. The variance that is associated with each year is now freely estimated, giving us the relative contribution of the four time points. The correlation between areas over time is assumed to be zero such that there is no relationship between the effect of the same area at different time points. This is unlikely to be so, and to an extent the cross-classified model therefore lacks realism. A comparison of equations (20) and (23) shows that both model C and model D require the estimation of four variances; the covariances in equation (23) are all constrained to be 0 and those in equation (20) are dependent on the variances, being constrained to give a correlation of 1 between all variance pairs.

### 3.5 Model E: correlated cross-classified model

We can combine the cross-classified model D and the unconstrained multiple-membership model C such that there will be correlations between areas at different time points. These correlations are induced by the assumed perfectly correlated effects of areas (from the multiple-membership model), which are additional to independent (uncorrelated) area effects (from the cross-classified model). Substantively, this model can be thought of as combining constant area effects that vary in magnitude according to the year or stage of the life course with area effects that represent the ways in which areas change over time (and are therefore uncorrelated). We now write 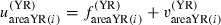
(24) with distributional assumptions given by expressions (18), (19) and (22). This means that 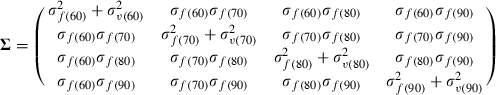
(25) where 
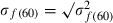
 etc. The variance of logit(*π*_*i*_) for person 1 in Table 1 is now given by 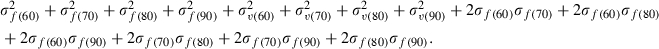


For person 5 the variance is 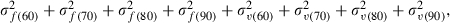 and the covariance between the two is 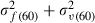. The variances are not constrained to be equal, meaning that we can estimate the proportion of the total variance that is associated with each time point, and the correlation between areas over time will be non-negative. There are now eight parameters to be estimated in **Σ**—the eight variances—since specification of these will determine the covariances. This compares with 10 parameters in the full multivariate model (see below). The potential gain in efficiency (through fewer parameters) comes at the expense of the constraint that the correlations in equation (25) cannot be negative; this gain will become larger as the dimension of **Σ**—i.e. the number of occasions on which we know where each individual lives—increases.

### 3.6 Model F: multivariate model

Finally, we consider a model in which we assume that the random effects have a full multivariate normal distribution. We adapt equation (2) by writing 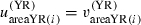, estimating a full covariance matrix such that 
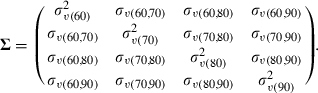
(26)

All the variances and covariances in this model are now freely estimated, subject to the usual constraints on a covariance matrix. For person 1 in Table 1 the variance of logit(*π*_*i*_) is 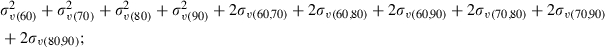 for person 5 the variance is 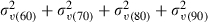 and the covariance is 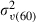.

## 4 Specification of priors

For all models we assume flat (improper) priors for the fixed effects of age 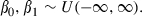


In general we assume uniform priors for the square root of the area variances; in this section we detail how these priors were operationalized. Throughout we have used uniform priors with a lower bound of 0 and an upper bound of 5; although not truly uninformative, the upper bound would correspond to a variance between areas of 25 on the log-odds scale. Assuming a threshold model, as described in Snijders and Bosker (1999), this would equate to a variance partition coefficient of 0.87; we felt it unlikely that the variances would be anywhere near this size. An alternative way of quantifying the magnitude of the variance in a multilevel logistic regression model is to use the median odds ratio that was proposed by Larsen and Merlo (2005); a variance of 25 implies that the median of the (ordered) odds ratios between individuals with identical covariates sampled randomly from two different areas would be an unfeasibly large 117.9.

### 4.1 Model A^(60)^, A^(70)^, A^(80)^, A^(90)^: separate two-level models including the effect of area of residence at a single year

We assume a normal distribution for the area level residuals with standard deviation *σ*_*v*(YR)_ to which we assign a uniform prior. For example for 1960 we have 
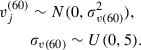
(27)

### 4.2 Model B: multiple-membership model

Again we assume a normal distribution for the area level residuals, this time with standard deviation *σ*_*f*_ to which we assign a uniform prior: 
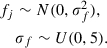
(28)

### 4.3 Model C: unconstrained multiple-membership model

We assume a standard normal *N*(0,1) distribution for the area level residuals and scale these by multiplying by the appropriate standard deviation *σ*_*f*(YR)_. This ensures that the correlations between the effects for one area at two different times are constrained to be 1. The *σ*_*f*(YR)_ are assigned uniform priors: 
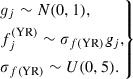
(29)

### 4.4 Model D: cross-classified model

We assume a normal distribution for the area level residuals with standard deviation *σ*_*v*(YR)_ to which we assign a uniform prior: 
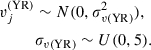
(30)

### 4.5 Model E: correlated cross-classified model

We use the prior specifications from Sections 4.3 and 4.4, combining the unconstrained multiple-membership model and the cross-classified model.

### 4.6 Model F: multivariate model

The priors that were used in the multivariate normal model differ from the priors that were listed in Sections 4.1–4.5 as implemented in WinBUGS (Spiegelhalter *et al.*, 2004). We assume a multivariate normal distribution for the four residuals (corresponding to the four censuses) for each area with inverse covariance matrix **Σ**^−1^ to which we assign a Wishart distribution: 
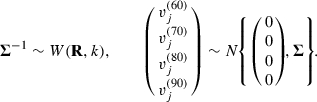
(31)

We chose the degrees of freedom for the Wishart distribution to be as small as possible (4, the rank of **Σ**^−1^). We considered three specifications of the scale matrix **R**: **I**, the identity matrix, 0.1**I** and 0.4**I**. **R** can be thought of as an estimate of the order of magnitude of *k***Σ** (Browne, 2005), and the choice of **R** can have a substantial effect on the results. Throughout we present only the results taking **R**=0.4**I**—a ‘good’ guess that the area variance that is associated with each year is approximately 0.1—but also discuss the results from the other two priors.

## 5 Results of modelling

Table 2 compares the estimated DIC for models A–F for each of the four cohorts. Under model A we see a clear preference for information on area of residence in 1990 for two of the cohorts (ages 30–39 and 50–59 years in 1990), for 1980 for one cohort (60–69 years) and no clear preference between 1980 and 1990 for the remaining cohort (40–49 years). For the cohorts of the three youngest ages (30–59 years) the DIC indicates a preference for the unconstrained multiple-membership model C over the (constrained) multiple-membership model B, with little to choose between the unconstrained multiple-membership model and the cross-classified model D. The correlated cross-classified model E proved to be an improvement on both multiple-membership models and the cross-classified model for these three cohorts. In the oldest ages cohort there was little to choose between the correlated cross-classified model E and the multiple-membership model B, with poorer fits evident for the unconstrained multiple-membership model C and cross-classified model D. Since the value of *p*_*D*_, the effective number of parameters, is dependent on the prior information (Spiegelhalter *et al.*, 2002), the DIC for the multivariate model F is also influenced by the change in prior compared with the other models but is presented here for completeness. *p*_*D*_ is calculated by subtracting the deviance of the posterior means, 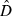, from the mean of the deviance. (The DIC also varies markedly according to the choice of the scale parameter of the Wishart distribution, **R**.)

**Table 2:** Comparison of 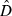, *p*_*D*_ and the DIC

*Model*	*Results for the following ages:*											
	*30–39 years*			*40–49 years*			*50–59 years*			*60–69 years*		
	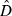	*p*_*D*_	*DIC*	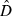	*p*_*D*_	*DIC*	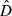	*p*_*D*_	*DIC*	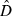	*p*_*D*_	*DIC*
A^(60)^, area 1960	2848	12.0	2872	4205	10.4	4226	6978	16.8	7012	18249	38.5	18326
A^(70)^, area 1970	2859	7.5	2874	4206	10.0	4226	6926	29.0	6984	18182	44.8	18271
A^(80)^, area 1980	2846	12.8	2871	4125	32.1	4189	6908	31.7	6971	18157	45.7	18249
A^(90)^, area 1990	2782	28.0	2838	4123	32.0	4187	6880	35.9	6952	18188	43.0	18274
B, multiple membership	2820	21.2	2862	4151	26.6	4204	6895	32.9	6961	18143	46.3	18236
C, unconstrained multiple membership	2782	30.5	2842	4108	38.4	4184	6874	40.5	6955	18133	53.6	18240
D, cross-classified	2754	43.3	2840	4077	53.4	4184	6842	56.1	6954	18087	79.6	18246
E, correlated cross-classified	2751	42.2	2835	4082	47.9	4178	6849	50.3	6949	18098	67.3	18233
F, multivariate	2737	54.7	2846	4061	65.6	4192	6806	77.1	6960	18047	99.8	18247

Table 3 shows the estimated proportion of the total area variation that is associated with each time point for models B–F together with 95% credible intervals. These proportions are estimated as the contribution of the relevant variance associated with one time point to the sum of the diagonal elements of **Σ**. For the general multivariate model (model F) with **Σ** given by equation (26), the proportion of the area variation that is associated with area of residence in 1960 would be 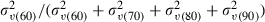; as such it ignores the covariances and strictly refers to those individuals who lived in four different areas. (The estimates of the variances, however, are derived from the entire data set and not just those who move between censuses.) The multiple-membership model with equal weights constrains this proportion to be 0.25 for each time point; we described this above as one of the weaknesses of such a model. The posterior means from the correlated cross-classified model (model E)—which had the lowest DIC of models A–E in Table 2—suggest that the area of residence in 1990 makes the largest single contribution to the variance for the three younger ages cohorts. The credible intervals indicate substantial overlap for these proportions; apart from those aged 30–39 years in 1990 it is difficult to be sure that the area of residence in 1990 was more important than the areas for each of the preceding time periods. The pattern is slightly different for the oldest ages cohort; the posterior means indicate heightened importance of the area of residence in 1980 but the total variation in mortality appears to be much more evenly distributed across the four time periods. The importance that is given to residence in 1990 for the three younger ages cohorts—and in 1980 for the oldest ages cohort—is supported by the findings from the models for single years (model A) in Table 2, for which the low values of the DIC suggest that these years provide the best fit. The multivariate priors generally provide estimates of this proportion that are much more evenly distributed over the four time points; for example, models C–E estimate the mean proportions that are associated with area of residence in 1990 for the youngest ages cohort to be between 0.68 (model E) and 0.89 (model C) compared with 0.48 for model F (and 0.39 or 0.56 when the alternative multivariate prior is used).

**Table 3:** Comparison of proportion of area variance associated with residence at each census

*Model*	*Age (years)*	*Results for 1960*		*Results for 1970*		*Results for 1980*		*Results for 1990*	
		*Proportion*	*95% credible interval*	*Proportion*	*95% credible interval*	*Proportion*	*95% credible interval*	*Proportion*	*95% credible interval*
B, multiple membership	30–39	0.25		0.25		0.25		0.25	
	40–49	0.25		0.25		0.25		0.25	
	50–59	0.25		0.25		0.25		0.25	
	60–69	0.25		0.25		0.25		0.25	
C, unconstrained multiple membership	30–39	0.04	(0.00,0.25)	0.02	(0.00,0.14)	0.04	(0.00,0.25)	0.89	(0.58,1.00)
	40–49	0.02	(0.00,0.13)	0.02	(0.00,0.14)	0.43	(0.01,0.92)	0.52	(0.04,0.95)
	50–59	0.04	(0.00,0.18)	0.11	(0.00,0.44)	0.14	(0.00,0.64)	0.71	(0.19,0.97)
	60–69	0.15	(0.01,0.38)	0.14	(0.00,0.52)	0.60	(0.12,0.92)	0.12	(0.00,0.51)
D, cross-classified	30–39	0.12	(0.00,0.40)	0.05	(0.00,0.24)	0.08	(0.00,0.33)	0.75	(0.42,0.97)
	40–49	0.04	(0.00,0.19)	0.04	(0.00,0.19)	0.42	(0.03,0.82)	0.50	(0.11,0.90)
	50–59	0.06	(0.00,0.23)	0.13	(0.00,0.42)	0.15	(0.00,0.51)	0.66	(0.27,0.94)
	60–69	0.07	(0.00,0.24)	0.26	(0.01,0.59)	0.48	(0.06,0.86)	0.19	(0.00,0.54)
E, correlated cross-classified	30–39	0.15	(0.01,0.41)	0.07	(0.00,0.26)	0.11	(0.00,0.36)	0.68	(0.37,0.92)
	40–49	0.06	(0.00,0.22)	0.06	(0.00,0.23)	0.40	(0.06,0.80)	0.48	(0.11,0.84)
	50–59	0.08	(0.00,0.28)	0.15	(0.01,0.44)	0.19	(0.01,0.55)	0.58	(0.20,0.88)
	60–69	0.13	(0.01,0.33)	0.24	(0.02,0.56)	0.40	(0.06,0.78)	0.23	(0.01,0.58)
F, multivariate	30–39	0.19	(0.07,0.39)	0.16	(0.06,0.34)	0.16	(0.05,0.34)	0.48	(0.24,0.72)
	40–49	0.15	(0.06,0.30)	0.16	(0.06,0.31)	0.33	(0.12,0.59)	0.37	(0.15,0.63)
	50–59	0.17	(0.07,0.32)	0.23	(0.10,0.41)	0.23	(0.10,0.42)	0.38	(0.17,0.61)
	60–69	0.17	(0.09,0.29)	0.26	(0.13,0.43)	0.31	(0.15,0.51)	0.26	(0.13,0.44)

Table 4 shows, for each cohort and under each of models B–F, the posterior means of the correlations between the area effects across the four census years implied by the model, i.e. from the estimated covariances and variances (above the diagonal) and the correlations between the posterior mean estimates of the area residuals (below the diagonal). For the general multivariate model (model F) the correlation between the area effects for 1960 and 1970 that is implied by equation (26) is 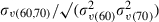. Under both multiple-membership models (models B and C) the correlations are constrained to be 1. In contrast the cross-classified model D models the area effects independently under the assumption that they are uncorrelated over time. The observed correlations between all pairs of residual sets for each cohort were positive under models D and E. Other notable features were that the correlations tended to be higher when they referred to years that were closer in time and tended to be higher for the older ages cohorts than for the younger ages cohorts. In all cases for model E the observed correlations between pairs of residual sets were higher than the implied correlations. In contrast many of the correlations—both modelled and observed—were negative for the multivariate model F, although the observed correlations did tend to become larger and were more likely to be positive as those in the cohorts aged and the proportion of deaths increased. It is possible that the negative modelled correlations—which were more abundant for the two alternative priors—at least in part reflect negative correlations in the joint posterior between the variances rather than the residuals, with a finite total area variance being split into four parts corresponding to the different time points.

**Table 4:** Modelled (above diagonal) and observed (below diagonal) correlations between area effects

*Model*	*Year*	*Results for the following ages and years:*															
		*30–39 years*				*40–49 years*				*50–59 years*				*60–69 years*			
		*1960*	*1970*	*1980*	*1990*	*1960*	*1970*	*1980*	*1990*	*1960*	*1970*	*1980*	*1990*	*1960*	*1970*	*1980*	*1990*
B, multiple membership	1960		1	1	1		1	1	1		1	1	1		1	1	1
	1970	1		1	1	1		1	1	1		1	1	1		1	1
	1980	1	1		1	1	1		1	1	1		1	1	1		1
	1990	1	1	1		1	1	1		1	1	1		1	1	1	
C, unconstrained multiple membership	1960		1	1	1		1	1	1		1	1	1		1	1	1
	1970	1		1	1	1		1	1	1		1	1	1		1	1
	1980	1	1		1	1	1		1	1	1		1	1	1		1
	1990	1	1	1		1	1	1		1	1	1		1	1	1	
D, cross-classified	1960		0	0	0		0	0	0		0	0	0		0	0	0
	1970	0.336		0	0	0.333		0	0	0.105		0	0	0.508		0	0
	1980	0.345	0.539		0	0.171	0.316		0	0.253	0.510		0	0.445	0.575		0
	1990	0.062	0.101	0.182		0.080	0.125	0.448		0.183	0.382	0.577		0.255	0.268	0.685	
E, correlated cross-classified	1960		0.267	0.309	0.197		0.286	0.324	0.280		0.286	0.308	0.318		0.441	0.588	0.379
	1970	0.666		0.309	0.206	0.634		0.331	0.283	0.584		0.294	0.318	0.869		0.420	0.257
	1980	0.687	0.812		0.224	0.687	0.763		0.330	0.700	0.781		0.311	0.918	0.853		0.369
	1990	0.331	0.450	0.465		0.597	0.634	0.712		0.648	0.720	0.805		0.761	0.647	0.863	
F, multivariate	1960		−0.265	0.092	−0.033		−0.137	−0.005	−0.023		−0.221	0.070	0.042		0.076	0.210	0.067
	1970	−0.219		−0.049	−0.103	0.002		−0.011	−0.117	−0.321		−0.107	0.065	0.490		0.037	−0.170
	1980	0.390	0.282		−0.070	0.085	0.163		0.235	0.254	0.226		−0.033	0.622	0.484		0.002
	1990	−0.014	−0.170	0.023		0.024	−0.088	0.667		0.154	0.336	0.479		0.294	−0.046	0.532	

Table 5 presents the posterior means of the variances between areas together with 95% credible intervals, under each of models B–F and for each cohort, for two distinct groups of individuals: those who moved from one area to another between censuses and lived in four different areas (movers—person 5 in Table 1) and those who lived in the same area at the time of all four censuses (stayers—person 1 in Table 1). The variances that were monitored were those described algebraically following the description of each model in Section 3. The movers comprised 6.8% of the total population, ranging from 2.1% in the oldest ages cohort to 11.0% of those aged 40–49 years in 1990. The stayers made up 22.8% of the total population, ranging from 14.1% among those aged 40–49 years in 1990 to 37.9% of those aged 60–69 years. All the variances are on a log-odds scale. For each model the variances consistently decrease with increasing cohort ages (and consequently with increasing mortality rates in the cohorts). The independence of the variances at different time points that were assumed under the cross-classified model D means

**Table 5:** Estimated variance of logit(*π*_*i*_) for movers and stayers

*Model*	*Ages (years)*	*Results for movers*		*Results for stayers*	
		*Variance*	*95% credible interval*	*Variance*	*95% credible interval*
B, multiple membership	30–39	0.067	(0.003,0.170)	0.268	(0.012,0.679)
	40–49	0.060	(0.018,0.127)	0.241	(0.071,0.508)
	50–59	0.045	(0.022,0.081)	0.182	(0.087,0.326)
	60–69	0.038	(0.021,0.063)	0.152	(0.086,0.251)
C, unconstrained multiple membership	30–39	0.253	(0.093,0.513)	0.457	(0.181,0.911)
	40–49	0.176	(0.073,0.344)	0.411	(0.177,0.781)
	50–59	0.092	(0.040,0.182)	0.219	(0.108,0.389)
	60–69	0.056	(0.029,0.101)	0.163	(0.091,0.270)
D, cross-classified	30–39	0.345	(0.152,0.634)	0.345	(0.152,0.634)
	40–49	0.290	(0.136,0.517)	0.290	(0.136,0.517)
	50–59	0.168	(0.086,0.286)	0.168	(0.086,0.286)
	60–69	0.113	(0.068,0.175)	0.113	(0.068,0.175)
E, correlated cross-classified	30–39	0.395	(0.181,0.708)	0.551	(0.249,1.030)
	40–49	0.286	(0.129,0.517)	0.440	(0.208,0.807)
	50–59	0.150	(0.071,0.268)	0.235	(0.122,0.407)
	60–69	0.088	(0.047,0.148)	0.170	(0.097,0.278)
F, multivariate	30–39	0.430	(0.240,0.709)	0.334	(0.131,0.685)
	40–49	0.319	(0.188,0.513)	0.325	(0.146,0.612)
	50–59	0.235	(0.146,0.374)	0.210	(0.113,0.359)
	60–69	0.158	(0.108,0.228)	0.168	(0.100,0.268)

that the variances are the same for the movers and stayers (and for any other migration pattern). In models B, C and E the positive correlation between area effects at different times meant that the variances in the contribution of areas to mortality were higher among those who had lived in the same area at all four censuses (and who therefore had had, we assume, continuous exposure to that area) than among those who had moved, albeit such differences were small relative to the credible intervals. The negative correlations between area effects at different times that are seen in Table 4 for model F meant that in two of the cohorts (ages 30–39 years and 50–59 years in 1990) the mean posterior total area variance was larger for movers than for stayers. For the other two cohorts the variance was larger for the stayers.

**Fig. 1:**
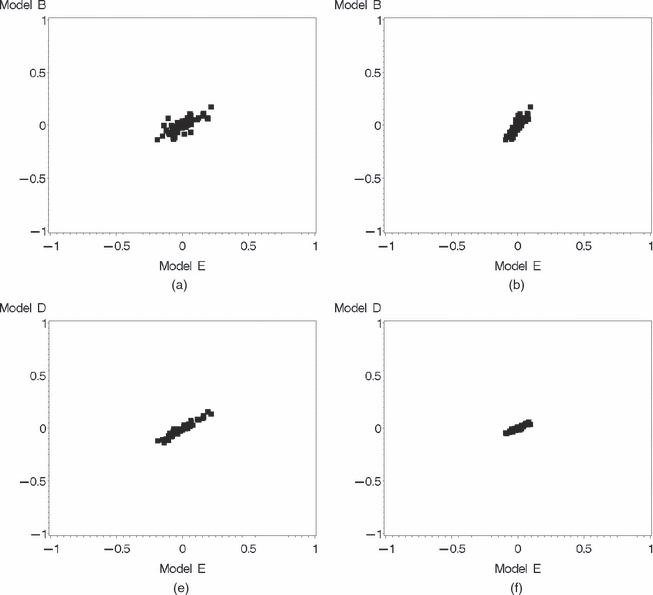

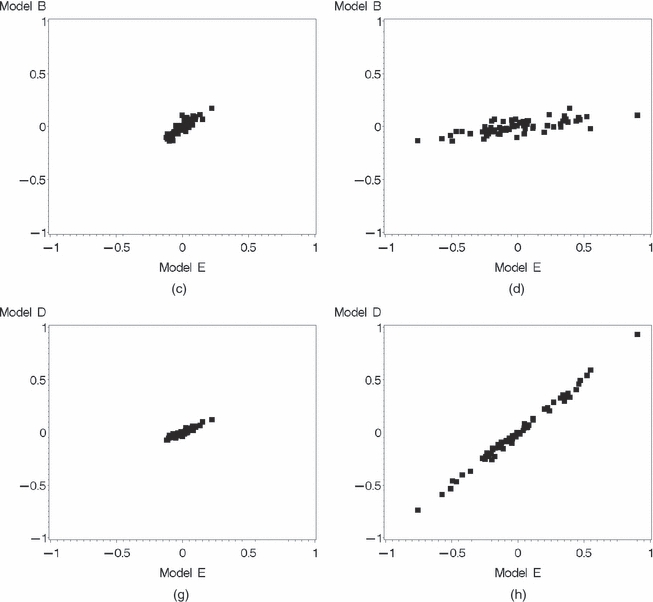
Comparison of residuals from (a)–(d) model B (multiple membership) and (e)–(h) model D (cross-classified) with model E (correlated cross-classified) associated with the four different years, ages 30–39 years in 1990: (a), (e) 1960; (b), (f) 1970; (c), (g) 1980; (d), (h) 1990

**Fig. 2:**
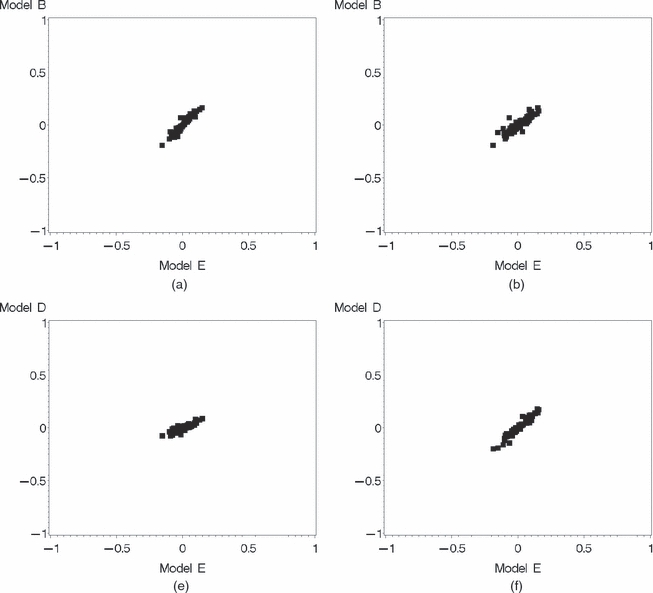

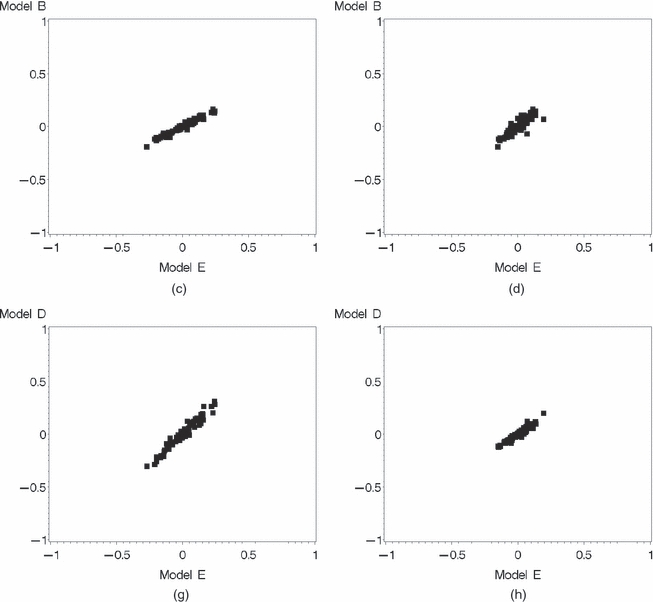
Comparison of residuals from (a)–(d) model B (multiple membership) and (e)–(h) model D (cross-classified) with model E (correlated cross-classified) associated with the four different years, ages 60–69 years, in 1990: (a), (e) 1960; (b), (f) 1970; (c), (g) 1980; (d), (h) 1990

The variance partition coefficient is a commonly used means of expressing the proportion of the total variance that is attributable to each level of a multilevel model. A variety of methods of estimating the variance partition coefficient have been proposed (Goldstein *et al.*, 2002); here we consider only that based on the assumption of a threshold model as described by Snijders and Bosker (1999), knowing that this ignores the effect of individual covariates but aware of the computational requirements of the simulation-based approach (Browne *et al.*, 2005). The variances for the correlated cross-classified model E in Table 5 suggest that in the oldest ages cohort 2.6% (95% credible interval 1.4–4.3%) of the total variance among movers and 4.9% (95% credible interval 2.9–7.8%) among stayers are due to the effects of the areas in which they have lived, with these figures increasing to 10.7% (95% credible interval 5.2–17.7%) and 14.3% (95% credible interval 7.0–23.8%) for movers and stayers respectively in the youngest ages cohort. Fig. 1 shows the extent of the correlation between composite area residuals at each time point 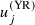 estimated under each of the multiple-membership model B and the cross-classified model D with the correlated cross-classified model E for the youngest ages cohort. The estimates under the different models are in general strongly correlated, but the estimates that were obtained under model D are closer to those for model E. Fig. 2 repeats these plots for the oldest ages cohort; although the correlations between the estimates made under different models at this age are not as strong as in the younger ages cohort, the absolute size of the area effects tends to be smaller at older ages (the variances in Table 5 tend to decrease as cohort ages increase). The degree of scatter along the axes in each figure is indicative of the magnitude of the area variance that is associated with that year. For the multiple-membership models the estimated area variance is the same in each year, whereas both the cross-classified and the correlated cross-classified models suggest a larger variance associated with the area of residence in 1990 for the youngest ages cohort and a more equal distribution of the variance in the oldest ages cohort (with a slight increase seen for 1980). In this sense these figures are illustrative of the proportions that are given in Table 3. The strong correlations mean that a ranking of the estimated area effects would not differ substantially according to the model chosen.

The standard deviations of the composite area effects at each census, 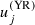, provide an indication of the certainty with which each can be estimated. The means of the posterior estimates of the standard deviations 
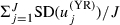
, which are obtained from the summary statistics of the composite residuals, are given in Table 6, for models B–F and for each cohort and time point. Also shown are the means of the posterior estimates of the standard deviations for stayers in each area. Since the estimates for model B are the same at each time point, it is difficult to make comparisons between this and the other models. The other models show a tendency for standard deviations to be larger for residence in 1990, with the exception of the oldest ages cohort for which standard deviations are greatest in 1980. In this sense the standard deviations clearly follow the pattern of the area variances (and, by implication, the area residuals themselves)—as the residual variance increases, so does the uncertainty that is associated with the estimate of each. Standard deviations for models D and E are broadly equivalent and larger than those for the unconstrained multiple-membership model C. Standard deviations for the multivariate model F reflect the more even distribution of the area variance across time points that is seen in Table 3. The mean standard deviations of the area estimates for ‘stayers’ are slightly larger under the correlated cross-classified model E than under the other models, again reflecting larger absolute values for the residuals.

**Table 6:** Mean standard deviations of composite area effects associated with residence at each census

*Model*	*Age (years)*	*Mean standard deviations for the following years:*				*Mean standard deviations for stayers*
		*1960*	*1970*	*1980*	*1990*	
B, multiple membership	30–39	0.109	0.109	0.109	0.109	0.435
	40–49	0.097	0.097	0.097	0.097	0.387
	50–59	0.077	0.077	0.077	0.077	0.307
	60–69	0.055	0.055	0.055	0.055	0.221
C, unconstrained multiple membership	30–39	0.081	0.057	0.079	0.362	0.510
	40–49	0.047	0.048	0.202	0.220	0.448
	50–59	0.044	0.070	0.080	0.182	0.318
	60–69	0.054	0.056	0.110	0.053	0.223
D, cross-classified	30–39	0.191	0.127	0.154	0.395	0.464
	40–49	0.101	0.100	0.292	0.305	0.400
	50–59	0.091	0.132	0.146	0.253	0.293
	60–69	0.079	0.141	0.177	0.127	0.202
E, correlated cross-classified	30–39	0.213	0.148	0.185	0.396	0.557
	40–49	0.116	0.115	0.272	0.287	0.464
	50–59	0.099	0.128	0.143	0.224	0.330
	60–69	0.080	0.114	0.135	0.116	0.227
F, multivariate	30–39	0.257	0.243	0.236	0.371	0.471
	40–49	0.192	0.198	0.266	0.278	0.426
	50–59	0.167	0.195	0.202	0.238	0.324
	60–69	0.123	0.159	0.171	0.164	0.231

The mean posterior estimates of the fixed parameters ***β*** and their standard deviations were similar for all models.

## 6 Conclusions

Comparing the fit of the various models to the data, as assessed by the DIC, suggested that the correlated cross-classified model provided an improvement over the other models considered for those aged 40–49 years in 1990. For the oldest ages cohort there was little to choose between that model and the multiple-membership model whereas for those aged 30–39 and 50–59 years in 1990 the simple model including only area of residence in 1990 proved comparable with the correlated cross-classified model. However, neither the multiple-membership model nor the models including only the area of residence at one time point had the ability to address our substantive research question about the relative importance of area of residence at each time point. The differences that were apparent between models suggested that those models that might be considered to be obvious alternatives to the correlated cross-classified approach that we propose—the multiple-membership and cross-classified models—may both tend to underestimate variances for certain groups of people. The results that were obtained for the model with a full multivariate normal prior on the covariance matrix were strongly influenced by the prior specification of the scale matrix; as such it was difficult to make direct comparisons with the other models although the results did appear to be broadly in line.

In this paper we have introduced an extension to the multiple-membership model by showing how weights may in effect be estimated empirically from the data as an alternative to an *a priori* specification. This is of particular importance in areas of research such as life course epidemiology in which the classifications are ordered sequentially. Living in area X in 1960 and area Y in 1990 may have a different effect on health from living in area Y in 1960 and area X in 1990 because area X may have changed between 1960 and 1990 or because 1990 may coincide with a more important (possibly just more recent) stage of the life course. The fit of the unconstrained multiple-membership model—as assessed by the DIC—proved similar to that for the cross-classified model. But, just as the cross-classified model makes the assumption that the area effects are uncorrelated over time, the unconstrained multiple-membership model still assumes that there is perfect correlation between area effects at one time point and another. The data suggested that area effects were positively correlated over time but with correlation less than 1. We have shown how such correlations between classifications can be modelled by combining the independent effects of the cross-classified model with the correlated effects of the (unconstrained) multiple-membership model. Such models have widespread potential as a modelling framework when repeated observations are made on the environment or context which influences individual outcomes.

The differences between the results that were provided by the various models were not large and did not materially affect the substantive conclusions that we drew—namely that the most recent areas of residence (1990 or, in the case of those aged 40–49 years in 1990, 1980 and 1990) were most influential in determining mortality for the youngest ages cohorts aged 30–59 years in 1990 whereas the oldest ages cohort showed more evidence of having accumulated risk from areas of residence across the life course. These findings are consistent with earlier findings based on these data in which cause-specific analyses have shown that there is a cumulative effect across the life course for chronic diseases such as coronary heart disease, chronic obstructive lung disease and smoking-related cancers, which are more common at older ages, and a critical period effect of more recent circumstances for violent and psychiatric causes of death, which are more common at younger ages. Such findings held when either individual level socio-economic life course factors (Næss *et al.*, 2004) or area influences (Næss *et al.*, 2008) were considered. Our observation that the correlations between area effects tended to be greater in the older ages cohorts may indicate that the contextual factors influencing health differ according to the age of the individual. The general differences in the causes of death at different ages may be the mechanism through which such different contextual factors exert their influence, with factors affecting violent and psychiatric deaths subject to greater changes over time (and hence the smaller correlations) than those area characteristics affecting chronic diseases. This paper provides the framework for subsequent detailed evaluation of the relative contribution of individual and area characteristics across the life course on mortality.
